# Gynecological and Obstetrical Society of Chicago

**Published:** 1891-09

**Authors:** 


### GYNECOLOGICAL AND OBSTETRICAL SOCIETY OF CHICAGO.

REGULAR MEETING, APRIL 17TH, 1891.

The President, W. M. JAGGARD, in the chair.

DR. T. J. WATKINS read a paper on

### LACERATION OF THE ANTERIOR VAGINAL WALL, AND ITS REPAIR.*

DR. HENRY T. BYFORD: The operation, as performed by Dr. Watkins, is, I think, a remarkably useful one. It happens that the same shape of denudation was hit upon by myself and put into the last edition of Byford on "Diseases of Women," viz., a vaginal strip taken from each sulcus or each urethral notch and carried back. At the same time I describe the method of catching in the fascia so as to draw up the edges and attach them, giving them fascial attachment. In case it becomes necessary to shorten the anterior vaginal wall antero-posteriorly, a transverse strip can be denuded between these two. The method of putting in these sutures is also illustrated. I have no doubt Dr. Watkins' whole method is original, and there is one part that makes it more efficient, viz., extending the denudation straight back to the cervix. I have operated enough times to have had a good deal of trouble, and have had to give a good deal of thought to the subject of curing anterior colpocele and cystocele, and I know that whatever procedure we adopt the

*See original article, page 385.

condition is apt to return. I have denuded a portion from either side, where the sulci run into the little notch on either side of the urethra, and have carried the threads up behind the symphysis and out through the abdominal wall. That was all right; there was a good cicatricial contraction. But after a while the anterior wall comes down from further up nearer the cervix, and the loose tissue commences to protrude below the place that I have fixed up so nicely. So it struck me that Dr. Watkins had hit the nail on the head when he went back and took all the lateral tissue off—didn't leave any to come out. We know that even in virgins the uterus will come down with the vagina, and I wonder whether, unless the doctor takes out a good, wide strip from each posterior sulcus clear back, the uterus, not being firmly attached, will not push it all down again. We know the fascia about the cervix is quite loose when the cervix is down or well forward. The fascia, that passes on either side of the cervix through the broad and sacro-uterine ligaments, makes good points of attachments when it is firm, but when it is relaxed there is nothing to fix the vagina to the other end. So I am anxious to know whether this is, in such cases, a permanent cure. I have often been rather discouraged and wondered what we could do. I have taken out a good deal of vagina in front and behind, and have often found, after a few months, as much tissue there as before, peeping out triumphantly.

DR. J. ALEXANDER LYONS: I had the pleasure of assisting Dr. Watkins in the first few of his operations on the anterior vaginal wall, and the immediate result was so beautiful, and, indeed, so far as we can at present find out, so permanent, I feel like saying it is the operation that should be adopted on nearly all occasions for the relief of the vesical symptoms he has enumerated.

I have made the denudations, as he directs, on three patients with good results; seven months have elapsed since my first operation, but in no case has there been a return of the vesical symptoms.

One of my cases was a patient on whom a friend of mine was doing trachelorrhaphy and perineorrhaphy. I noticed there was also a marked prolapse of the anterior vaginal wall, and suggested the Watkins operation. The doctor kindly allowed me to perform it; primary union followed, and the vesical symptoms, which the patient complained of before the operation, were relieved, although the perineum did not unite. I suppose union failed in the perineum because of septic discharges lying there, which would not be so likely to disturb the anterior wall.

DR. F. H. MARTIN: I would like to say a few words on this subject. I came here laboring under a slight delusion, as my impression, from the statement in the notice of the meeting, was that the operation was not only for laceration of the vagina, but for relaxation of the walls from any cause. The anterior vaginal wall is a hypothenuse of a right-angled triangle, the base of the triangle being represented by the pubes, the other side of the triangle by tissues from the superior margin of the pubes near the spine or in the region of the insertion of the round ligament to a point near where the anterior vaginal wall joins the cervix. The side of the triangle formed by the anterior wall of the vagina is considerably longer than the other long side, and forms with the utero-sacral ligaments a strong support spanning the entire pelvis. In case of pressure from above, long continued, the recto-uterine ligaments stretch, and the upper angle or the apex of the triangle is lowered, thereby causing necessarily a sagging in the anterior vaginal wall. In those cases where there are no lacerations of the vagina, and in cases of cystocele where childbirth may never have taken place, or, in a word, in old hospital cases, occurring in scrubwomen, washerwomen, etc., caused from pressure of the abdominal walls on this support across the pelvis, causing curvature in the anterior vaginal wall and the crowding of the bladder after it, operative interference is required as certainly as in those caused by lacerations. The anterior vaginal wall is made up of the vaginal layer of mucous membrane, which is thick and develops in case of pregnancy, and sometimes remains hypertrophied or in a state of subinvolution. Next to this we have the muscular coat, which is a continuation of the middle coat of the uterus, which of necessity becomes hypertrophied in cases of pregnancy, and which may remain in a state of subinvolution afterward, but which in its normal condition lies in folds, so that it throws the mucous membrane into transverse rugæ. The inner coat is composed of connective tissue, and is directly connected with the fascia of the pelvis, as stated by Dr. Watkins. Now, if we have a laceration of the anterior vaginal wall causing a cystocele, if we have subinvolution of the anterior vaginal wall, or if we have the condition of stretching that I have mentioned, in which we get hospital prolapse, we should perform almost an identical operation. That operation should not have for its object the removal of tissue, because ordinarily we have no more tissue than the Lord put there. If hypertrophy has occurred, it is not necessary to remove the tissue, but by properly distributing it we can restore the parts to a proportionate condition of health, and involution will do the rest. I have been very much surprised this evening to find that Dr. Watkins has described, in many respects, an operation which I have performed for these three conditions for a number of years, but in which I think I have adopted one or two procedures of advantage which he has not mentioned. I have made a drawing, and here I present a model out of a glove to represent the method of operating. In the first place, I have recognized the fact that the deep tissue should be reached instead of the muscular and mucous coats alone. I have made an elliptical denudation of the mucous membrane, very narrow in the same location that Dr. Watkins has described; after making this denudation, I dissect under the edges of the undenuded tissues laterally, so as to be able to get a larger freshened surface than the narrow denuded surface would allow otherwise, and be able to reach the deep fascia on either side with my buried stitches. I seek, in my operation, to narrow the vagina laterally by narrowing the fascial coat, while I care for the superabundant muscular coat of the vagina by throwing it into its original condition of transverse folds. When this is accomplished by means of the peculiar insertions of the buried catgut, the edges of the mucous membrane are in apposition, ready to be sutured with simple superficial stitches. I accomplish the results described by a peculiar method of inserting the buried stitches. Each portion of catgut is inserted so as to have four points of traction, each point of insertion constituting one of these points; and when the quadruple stitch is tied it brings the four points in apposition. The upper two of the four insertions are deep and include the fascia; the lower two are more superficial, including the muscular coat alone.

DR. KARL SANDBERG: I was very favorably impressed with the operation devised by Dr. Watkins, and certainly consider it a great improvement upon the earlier methods of performing colporrhaphy—the old method, I might say, of making denudation in the middle and always of a certain form and with the sutures tied together in the middle. Dr. Watkins has devised this operation with the idea of the fascia being lacerated along the sulcus of one or both sides, all the way from the uterus down to the urethra. There is a little doubt in my mind whether this operation could not be still further improved; it seems to me to be schematized a little too much. I noticed in the list that out of twenty-three cases twenty-one were bilateral. I believe that by further experience this bilateral operation will be limited to fewer cases. I think that while the operator in the beginning may be afraid of overlooking any lacerations, and to be safe makes the operation extensive, the experienced eye or finger will be able to detect just where the laceration is and limit the operation to this point. It seems a little unreasonable that twenty-one of these twenty-three cases should have a laceration on both sides of the vagina extending all the way from the cervix uteri to the urethra. In regard to denudation, I should agree with Dr. Martin that it is absolutely unnecessary to remove any part of the mucous membrane; there is no superfluous mucous membrane; there is nothing to be removed. If we only make a vaginal incision and dissect up a little to each side, we will find the separated ends of the muscle, and we can bring them together and thus make the operation easier. It is reasonable to suppose that as well a small laceration of the cervix uteri may cause a subinvolution, or rather loss of contractile power of the whole organ; so also a subinvolution of the vagina or pelvic floor may be caused by a laceration of only a small part of the same. If this supposition is right, then, in order to remedy the trouble, it would be necessary only to find this place and bring the lacerated parts together. If we can only develop our diagnostic faculties so far that we can put our finger on the spot and say, "There is where the laceration has occurred; this is the direction and that is the extension of the same," then, and only then, can we expect to perform a colporrhaphy intelligently, and until we reach this point we will be making extensive and multiple operations; so be sure to take in every possible laceration, and do not attempt to remove superabundant tissue that does not exist. There is undoubtedly in the matter of colporrhaphy a vast field for research yet, and while Dr. Watkins' operation is certainly a help in the right direction, I think there is still room for improvement.

DR. BANGA: I would like to ask Dr. Watkins how long after the operation he had seen the patients when he marked them down as cured. I would also ask whether, in preventing prolapse of the anterior wall, he considers emptying of the bladder during parturition as apt to prevent it; and I would like to know whether he has the urine drawn after the patient is put back in bed; also whether he puts the patient in prone position or allows her to lie on the back.

DR. T. J. WATKINS, in closing the discussion, said: The greater frequency of cystocele among workingwomen is due entirely to their mode of life. After confinement they are unable to take the time of rest necessary for involution. The character of their work also tends to produce cystocele. The more or less continued tension on the anterior vaginal wall excites a plastic exudate which bathes, softens the connective tissue and permits it to stretch.

The amount of tissue which should be removed in operations upon the vaginal canal is important. In order to get deep and firm union it is necessary to either fold the tissues upon themselves, or to bring them together by the method of flap-splitting. I do not see how this result can be obtained simply by sutures, as suggested by Drs. Martin and Sandberg.

The removal of the amount of mucous membrane suggested in this operation cannot be harmful, for the vaginal mucous membrane will stretch to almost any extent, as illustrated in complete prolapse of the uterus.

Dr. Martin evidently mistakes the object of the operation. The greatest objection to the median operation is that it shortens the anterior vaginal wall. This wall should be from two and a half to three inches in length, and the nearer we can keep the cervix to the sacrum the better will be the result.

In the cases which I have reported as cured, this term applies to the relief of symptoms and the removal of the mechanical indication for the operation.

I did not speak of the emptying of the bladder during labor, because it is a well established obstetric aphorism.

During the last two years I have paid particular attention to the prevention of the engagement of the anterior vaginal wall between the head and the pubes, and have never experienced any difficulty in accomplishing this.

I have avoided the use of the catheter in these cases as far as possible, on account of the risk of cystitis.

I have hitherto paid little attention to the patient's position during convalescence; the position suggested by Dr. Banga would probably diminish the tension on some of the sutures.

The denudation about the urethra is similar to that recommended by Dr. Byford.

When cystocele occurs in a virgin it is due to the constant tension and the plastic exudation as described above. Of course, the causes for the constant tension are various and often hard to determine. When the anterior vaginal wall is restored it is most essential to restore the proper direction of the canal, because it partially relieves the anterior vaginal wall from the weight of the uterus; that is, when the vaginal canal has its normal direction, intra-abdominal pressure is lateral; when the posterior vaginal wall is torn, intra-abdominal pressure is largely in the direction of the canal.

Dr. Sandberg made a slight mistake as to the location of the laceration. Two cases of unilateral laceration were reported.

